# Evolving capabilities of computed tomography imaging for transcatheter valvular heart interventions – new opportunities for precision medicine

**DOI:** 10.1007/s10554-024-03247-z

**Published:** 2024-09-30

**Authors:** Vitaliy Androshchuk, Natalie Montarello, Nishant Lahoti, Samuel Joseph Hill, Can Zhou, Tiffany Patterson, Simon Redwood, Steven Niederer, Pablo Lamata, Adelaide De Vecchi, Ronak Rajani

**Affiliations:** 1 School of Cardiovascular Medicine & Sciences, Faculty of Life Sciences & Medicine, King’s College London, London, UK; 2 Cardiovascular Department, St Thomas’ Hospital, King’s College London, London, UK; 3 School of Biomedical Engineering and Imaging Sciences, Faculty of Life Sciences & Medicine, King’s College London, London, UK; 4 Guy’s & St Thomas’ NHS Foundation Trust, King’s College London, St Thomas’ Hospital, The Reyne Institute, 4th Floor, Lambeth Wing, London, SE1 7EH UK

**Keywords:** Computed tomography, Transcatheter valvular intervention

## Abstract

The last decade has witnessed a substantial growth in percutaneous treatment options for heart valve disease. The development in these innovative therapies has been mirrored by advances in multi-detector computed tomography (MDCT). MDCT plays a central role in obtaining detailed pre-procedural anatomical information, helping to inform clinical decisions surrounding procedural planning, improve clinical outcomes and prevent potential complications. Improvements in MDCT image acquisition and processing techniques have led to increased application of advanced analytics in routine clinical care. Workflow implementation of patient-specific computational modeling, fluid dynamics, 3D printing, extended reality, extracellular volume mapping and artificial intelligence are shaping the landscape for delivering patient-specific care. This review will provide an insight of key innovations in the field of MDCT for planning transcatheter heart valve interventions.

## Introduction

Early transcatheter therapies were largely guided by echocardiographic techniques, which were used to not only establish a diagnosis but also to plan for subsequent procedures. As multi-detector cardiac computed tomography (MDCT) scanner technology evolved and its accessibility became more widespread, it rapidly established itself as the new gold-standard technique for pre-procedural planning for many valvular interventions [1]. Despite its ability to provide access to high resolution isotropic imaging and sub 0.5 mm resolution, MDCT has had to overcome its own inherent challenges to facilitate the growth of these procedures [2]. 

Firstly, cardiac MDCT is a far newer imaging modality than echocardiography and this has required training of cardiologists in what is otherwise a radiology driven imaging technique. The manipulation of datasets and the use of multiplanar reformatted images is a time-consuming process that requires dedicated training to reduce inter-observer and intra-observer variability [3]. This is particularly relevant for identification of subtleties associated with increased risk, which if missed, can lead to incorrect device sizing, suboptimal patient outcomes and increased co-morbidity. A lack of experience and access to imaging training remains a significant barrier to transcatheter therapy implementation beyond highly specialised centres, where the demand for treatment often outstrips capacity. Efficiencies in imaging planning and computer-assisted processing techniques are likely to enable improved access to care and reduce health inequalities across the globe. Additionally, as the spectrum of transcatheter devices for valvular interventions increases, so will the requirements for imaging processing [4, 5]. Whereas MDCT for transcatheter aortic valve replacement (TAVR) planning has largely become standardized, transcatheter mitral and tricuspid valve replacement (TMVR and TTVR) planning requires far more detailed analysis across the entire cardiac cycle to predict a successful outcome. This includes an assessment of (1) regurgitation aetiology, (2) annulus geometry and size, (3) biventricular morphology and function, (4) vascular access, (5) physiological ventricular-vascular coupling and importantly (6) device/host interactions. Finally, these increasing demands have been accompanied by an exponential growth in computer processing power, software and hardware development, and the introduction of artificial intelligence (AI) that was largely in its infancy a decade ago. Structural cardiac imagers should now not only be proficient in multimodality cardiac imaging but also the new language of medical bioengineering, which promises efficiency, reliable clinical outcomes, precision-based medicine and a true globalisation in structural heart valve treatments [6]. 

The principal purpose of this review is to detail some of the new techniques of biomedical engineering that are being applied to MDCT in the structural valvular disease domain. We hope that this will familiarise readers with the rapidly evolving capabilities of MDCT in relation to patient-specific modelling, computational flow dynamics, three-dimensional (3D) printing, AI and tissue characterisation, with examples that demonstrate the real-world impact of these rapidly evolving technologies.

## The strengths of computed tomography in structural cardiac intervention

**Fig. 1:**
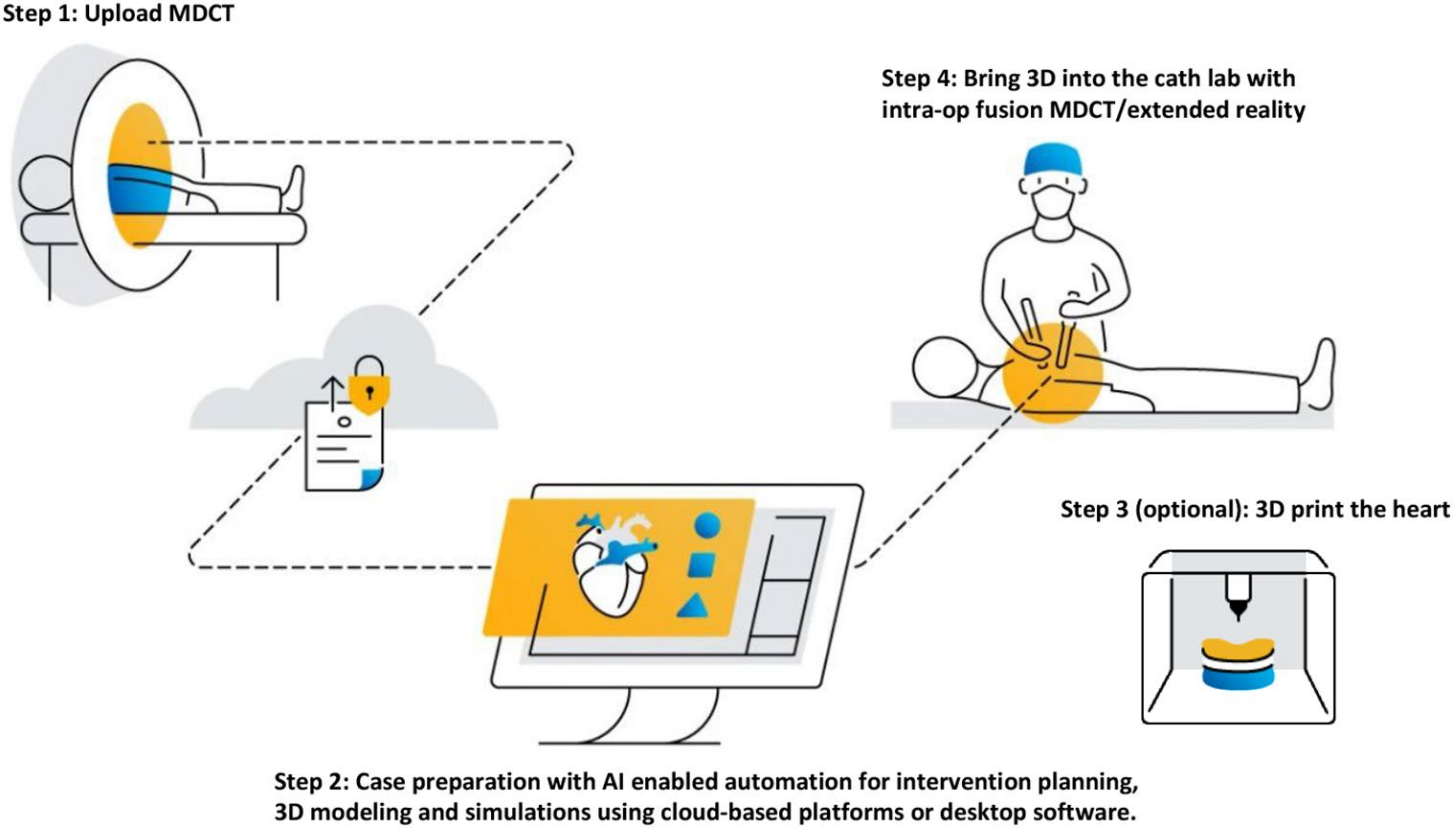
The stepwise workflow for advanced MDCT analysis. Patient MDCT images are securely uploaded into the online platform. The segmentation and 3D analysis of the case is performed using AI-enabled automation, which can be reviewed interactively online or using onsite desktop software package. If requested, a 3D model can be printed. The case plan can be brought into the cath lab for projection on a screen or alternatively, fusion and/or extended reality can be used. (Mimics Enlight, Materialise, Leuven, Belgium) (AI: artificial intelligence; MDCT: multi-detector computed tomography; 3D: three dimensional)

Comprehensive cardiac MDCT assessment is crucial in the treatment of valvular conditions with transcatheter interventions. Detailed anatomical evaluation using MDCT is pivotal for clinical decision-making regarding technical peri-procedural planning and post-procedural follow-up [7]. The increased use of MDCT for these indications has largely been driven by advances in scanner technology [8]. MDCT spatial resolution is superior to echocardiography and cardiac magnetic resonance (CMR) and offers the distinct advantage of isotropic imaging whereby the image dataset may be reconstructed in any plane without the loss of spatial resolution [9]. These properties make MDCT an ideal imaging modality for biomedical engineering applications and automated segmentation, 3D printing, computational modeling and AI techniques [10]. Computational modeling using MDCT can quantify the stress and deformation of myocardial tissues and characterise the intracardiac blood flow patterns, which can be used to assess the haemodynamic impact of interventions [11, 12]. The development of AI image analysis platforms and cloud-based clinical workflows has offered the promise of economical access and scalability for automatic analysis of large datasets [13]. These advantages and widespread accessibility, have enabled MDCT to position itself as the first-line imaging modality in the field of transcatheter valvular interventions (Fig. 1) [14]. Although this review is focused on MDCT, this does not detract from the importance of adopting the multimodality imaging approach to patients with heart valve diseases [15]. 

## Computed tomography imaging requirement for computational and physical modeling

The essential prerequisites for creating representative MDCT-based virtual simulations are good quality volumetric data with high spatial, temporal and contrast resolution, and accurate image segmentation. The precise specifications of the imaging protocols vary depending on the planned procedure and the scanner used [16]. In general, a clear depiction of the relevant structures is achieved with an ECG-gated and contrast-enhanced MDCT acquisition using preferably only moderate tube current modulation to maintain image quality throughout the cardiac cycle. In patients with primarily mitral or tricuspid valve pathology, low-dose negative chronotropic/inotropic agents are generally well tolerated and could be considered in situations where the baseline heart rate exceeds 100 bpm [17]. The timing of the contrast injection at 4–6 ml/s and the saline/contrast flush is determined by the cardiac structure being examined. The image reconstructions should be performed throughout the entire cardiac cycle at 5–10% increments of the R-R interval with a slice thickness < 1 mm. Image segmentation may then be applied to the selected phases in the cardiac cycle depending on the planned intervention and the cardiac region of interest.

Image segmentation refers to the conversion of the volumetric MDCT images into a digital 3D model with the desired heart chambers and great vessels [10]. There are several dedicated commercial and free semi-automated segmentation packages available, which are generally based on intensity thresholding and region growing [18–21]. Manual adjustment of the segmentations may be required if the delineation of the cardiac structure boundaries appears visually inaccurate. More recently, AI methodology has been applied to image segmentation with promising results [22]. Once the virtual 3D cardiac model is finalised, implantation of cardiac devices can be simulated with a range of clinical applications for transcatheter procedures. Alternatively, digital anatomic models can be saved in stereolithography (STL) file formats with the surface mesh information for 3D printing.

## Patient-specific simulations and computational modeling

Most commercial workstations can process MDCT data for planning transcatheter valvular interventions. Fundamentally, this requires access to multiplanar reformatting and automated or semi-automated image segmentation. Few platforms also offer the ability to perform simulations by deploying transcatheter devices virtually into the MDCT target zones. This involves using either a true stereolithographic image of the intended device size and type (provided by the device manufacturer) or a representative cylinder/shape of the desired valve (provided by the software vendor). The principal purpose of this analysis is to visualise the device within the patient’s anatomy and to assess a projected geometric outcome. Although undoubtedly valuable, the major limitations of this method are that it assumes device non-deformity through the cardiac cycle and largely ignores the mechanical stresses/interactions between the device and myocardial tissues.

Over recent years, personalised computer models of blood flow and interaction between flow and structure have emerged as a powerful tool to understand and predict pathophysiological changes in cardiac valvular diseases. These techniques are generally based on finite element modeling (FEM) or finite volume methods and work by solving first principles equations governing the behaviour of solids and fluids, resulting in biophysically accurate representation of patient-specific organ function [23]. Different types of models exist and can be broadly classified into three categories. Computational fluid dynamics (CFD) models allow simulation of blood flow in vessels and organs, revealing key haemodynamic markers, such as pressures or shear stresses, which are not directly available on routine non-invasive imaging modalities [24, 25]. CFD models can also be coupled to sophisticated rheological models of blood flow to simulate biochemical processes such as device thrombus formation [26, 27]. Personalised FEM-based models can simulate the mechanical impact of virtual valvular interventions, allowing for prediction of strains and contact forces during device deployment [28]. Finally, computer models of fluid-structure interaction (FSI) can simulate coupled dynamics of blood flow and solid mechanics, and their mutual effects [29, 30]. 

Regardless of the specific approach, these computer models are grounded in biophysical laws and offer the possibility to understand cause-effect relationships between physiological metrics during disease progression and after treatment. More recently, the technical approach has been refined by integrating patient-specific computer models with advanced machine learning technologies. This represents a significant leap forward in the realm of cardiac interventions: enabling significant acceleration of the model predictions within the Digital Twin paradigm for healthcare, which aims to integrate the medical imaging and clinical information to simulate relevant physiological processes in real time [31, 32]. This approach promises to enhance treatment outcomes, minimise procedural risks and pave the way for a new era of precision medicine in cardiovascular care. However, although these computational techniques offer substantial enhancements to standard image post-processing, they are costly and require specialist off-site post processing with the assistance from industry or academic partners.

### Planning for transcatheter aortic valve replacement

Computational modeling with FEM may have benefits in planning TAVR in patients with challenging anatomy. Due to the large variability in the aortic root shape, calcification burden and aortic valve morphology, incomplete or non-uniform TAVR frame expansion may lead to residual paravalvular leak (PVL). FEM can inform the expected sealing behaviour of TAVR prostheses to predict PVL in patients with either bicuspid or tricuspid aortic valve stenosis [33, 34]. The severity of residual PVL can then be assessed under various clinical scenarios, including using different device shapes, sizes and positions. The additional information derived from patient-specific computer models can assist with clinical decisions for treatment planning, such as the target depth of implantation, thereby improving TAVR device performance [35]. Heavily calcified or small aortic annuli pose additional challenges in terms of TAVR-induced conduction abnormalities. Modeling takes into consideration device-anatomy interactions and can predict areas of increased contact pressure on the region of the conduction system after TAVR implantation, which can identify patients at increased risk for permanent pacemaker implantation [36, 37].

**Fig. 2:**
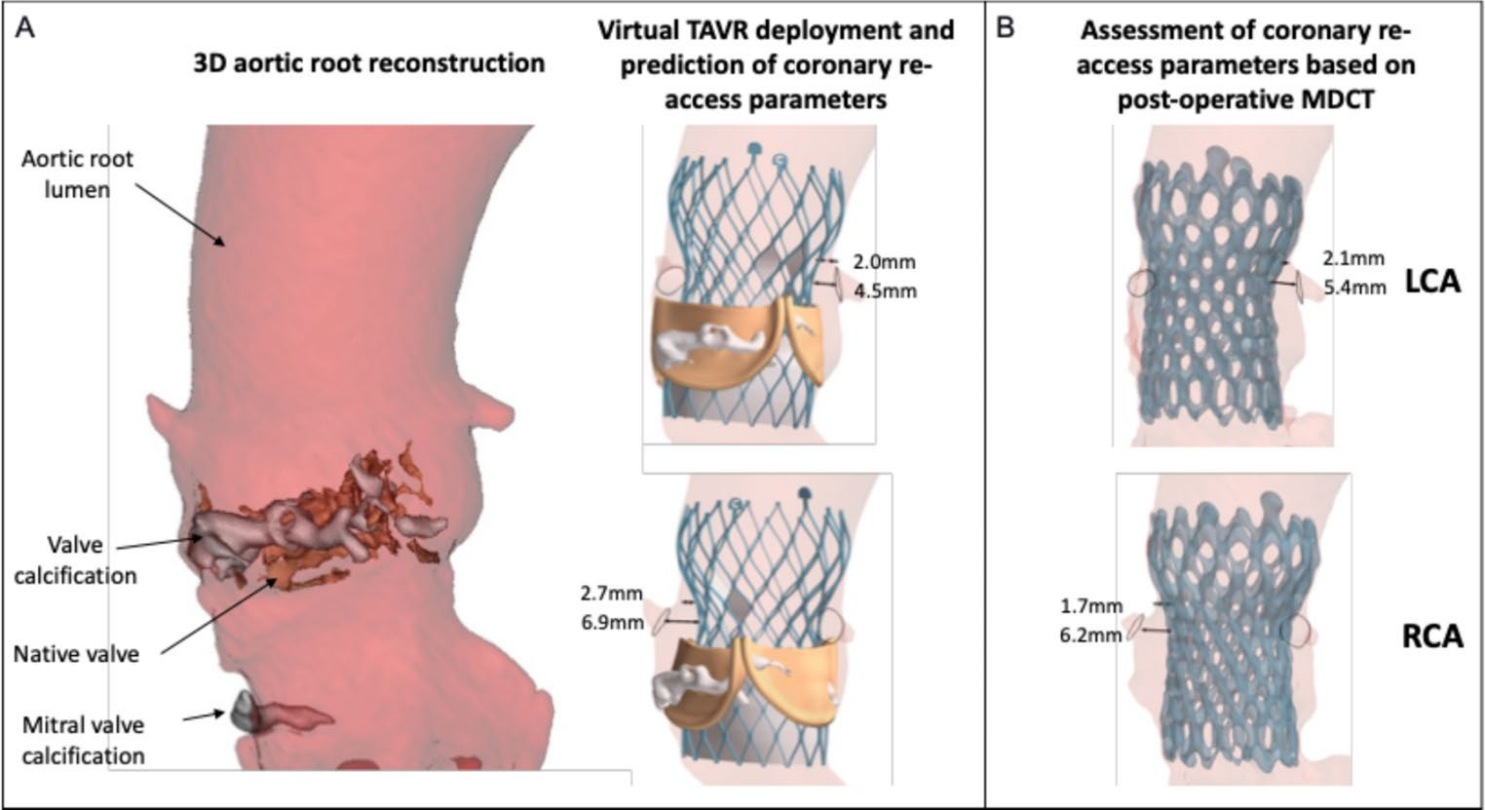
Visualisation of the finite element model for TAVR planning to predict re-access parameters for left and right coronary artery. **A**) Computer modeling workflow for virtual TAVR device deployment within the reconstructed aortic root, which is used to predict the valve-to-coronary and valve-to-STJ distances. **B**) Comparison with actual valve-to-coronary and valve-to-STJ distances achieved after TAVR on postoperative MDCT images. (FEops HEARTguide, Ghent, Belgium) (LCA: left coronary artery; MDCT: multi-detector computed tomography; RCA: right coronary artery; STJ: sinotubular junction; TAVR: transcatheter aortic valve replacement.)

Coronary artery re-access after TAVR can be challenging in a proportion of patients with bicuspid and tricuspid aortic valves [38]. Difficulties with coronary re-access after TAVR can arise because of commissural misalignment, narrow sinuses of Valsalva and the upward displacement of bulky native aortic valve leaflets [39–41]. This is a major concern for long-term management due to the expanding indications of TAVR to younger patients with longer life expectancy, where the need for future coronary angiography or intervention is likely to be higher. Modeling the expansion of a virtual TAVR prostheses inside a patient’s anatomy that includes annular and leaflet calcification can provide relevant anatomical parameters and ideal fluoroscopic projections to improve future coronary accessibility (Fig. 2). This can help interventionalists to perform patient-specific pre-procedural planning to identify the optimal type, size, position and depth of the final TAVR device to minimise the risk of unfavourable coronary re-access [42]. Other examples of MDCT-based model applications include prediction of TAVR outcomes in bicuspid aortic valve stenosis, and risk estimation of prosthetic leaflet thrombosis and coronary obstruction following TAVR [43–45]. Collectively, these findings demonstrate the feasibility and clinical utility of computer modeling to improve the safety, efficacy and clinical outcomes after TAVR, with further evaluation required to validate this technology for the lifetime management of valvular heart disease.

### Planning for transcatheter mitral and tricuspid valve interventions

**Fig. 3:**
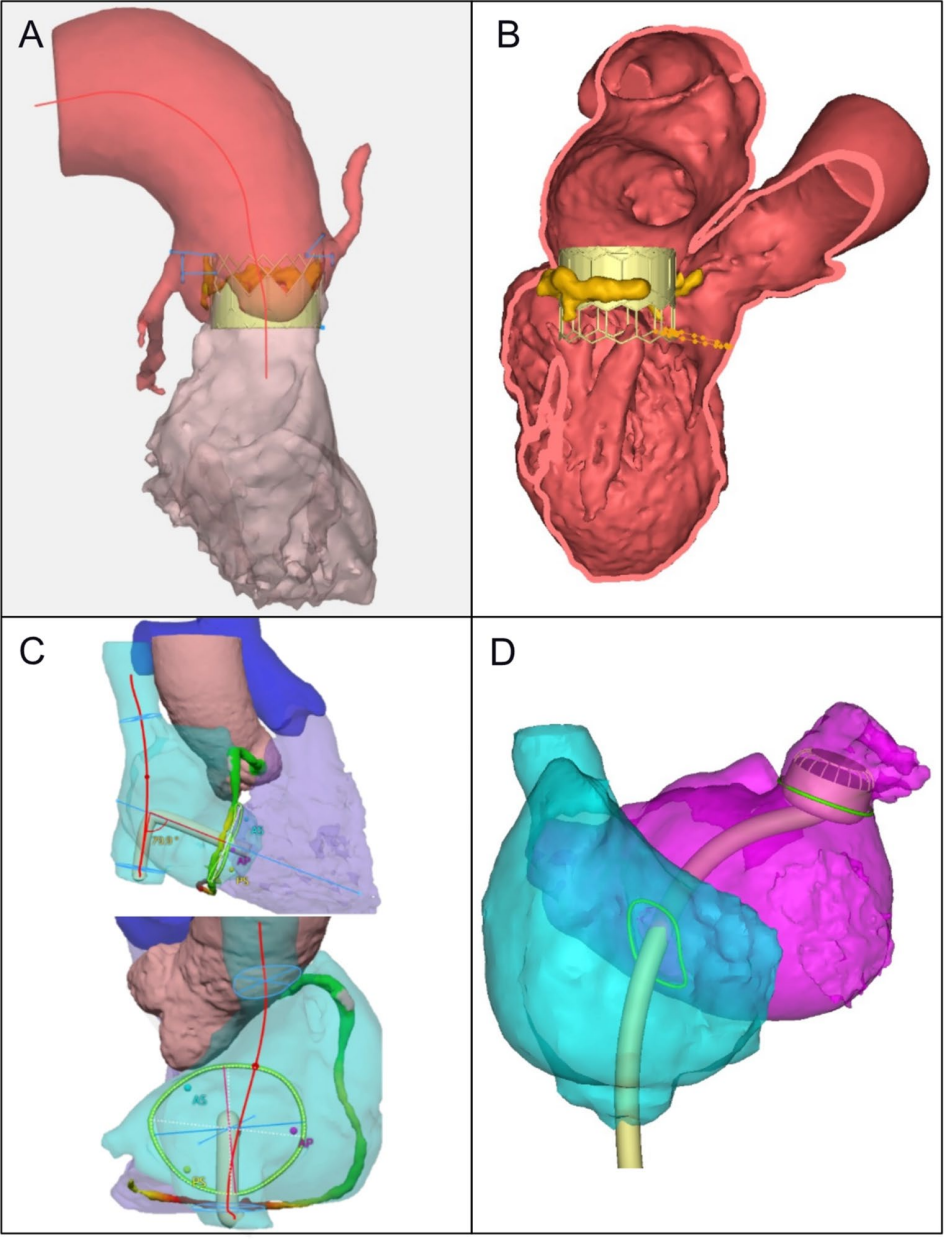
Illustration of patient-specific analysis and simulations for planning structural heart interventions using MDCT. **A**) Simulated TAVR implantation and assessment of risk for coronary occlusion and challenging coronary re-access. **B**) TMVR in MAC planning with automated neo-LVOT assessment. **C**) Delivery access planning for TTVR and assessment of relationships to adjacent right coronary artery. **D**) Planning of left atrial appendage occlusion, demonstrating catheter delivery pathway. (Mimics Enlight, Materialise, Leuven, Belgium). (MAC: mitral annular calcification; MDCT: multi-detector computed tomography; TAVR: transcatheter aortic valve replacement; TMVR: transcatheter mitral valve implantation; TTVR: transcatheter tricuspid valve implantation.)

Simulating the virtual deployment of TAVR devices provides clinicians with a visual perception of the interaction between the chosen prosthesis and native aortic root structures (Fig. 3a). In comparison with the tubular and planar shape of the aortic valve apparatus, the structure of the mitral and tricuspid valves is more complex, with asymmetrical and saddle-shaped annulus morphology. These show a dynamic response to myocardial contraction and undergo substantial changes in size and geometry during the cardiac cycle [46, 47]. As a result, the planning of transcatheter interventions for defects affecting these valves is more challenging. This necessitates a thorough understanding of the atrio-ventricular valve anatomy and geometry, with MDCT playing a crucial role in defining patient suitability for current transcatheter-directed mitral and tricuspid valve replacement therapies.

The proximity between the anterior mitral valve leaflet and the left ventricular outflow tract (LVOT) poses important anatomical challenges for TMVR. Implantation of a prosthesis into the mitral position can cause septal displacement of the anterior mitral valve leaflet, increasing the risk of LVOT obstruction [48]. This can lead to death, conversion to surgery, emergency reintervention or chronic adverse haemodynamics characterised by left ventricular remodelling and failure [49]. To enhance patient safety and procedural efficacy, the elongation of the native LVOT after TMVR, also referred to neo-LVOT, can be evaluated using different virtual valve sizes, designs and implantation heights (Fig. 3B) [50]. Currently, standard evaluation is largely simulation-based with the virtual implantation of the intended device onto the MDCT dataset, followed by the geometric measurement of the neo-LVOT area at its smallest. Previous studies demonstrated good agreement and low intra-observer and inter-observer variability between predicted and post-procedural neo-LVOT by manual planimetry on MDCT images [48]. 

**Fig. 4:**
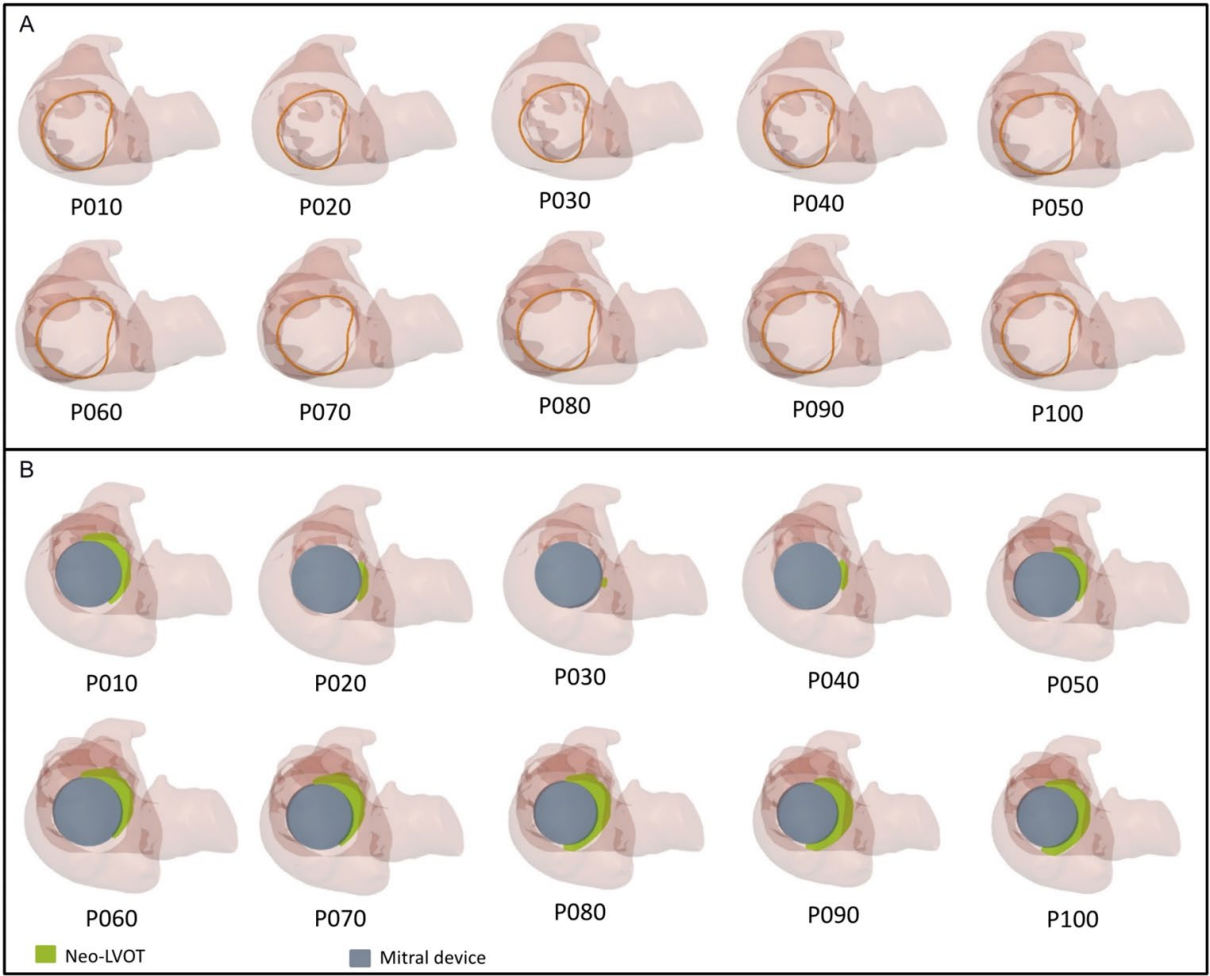
Creation of a full cardiac cycle MDCT-derived computational model for TMVR planning. **A**) Automated mitral annulus detection over the full cardiac cycle for TMVR planning. **B**) TMVR neo-LVOT prediction over the full cardiac cycle. (FEops HEARTguide, Ghent, Belgium) (MDCT: multi-detector computed tomography; TMVR: transcatheter mitral valve replacement.)

**Fig. 5:**
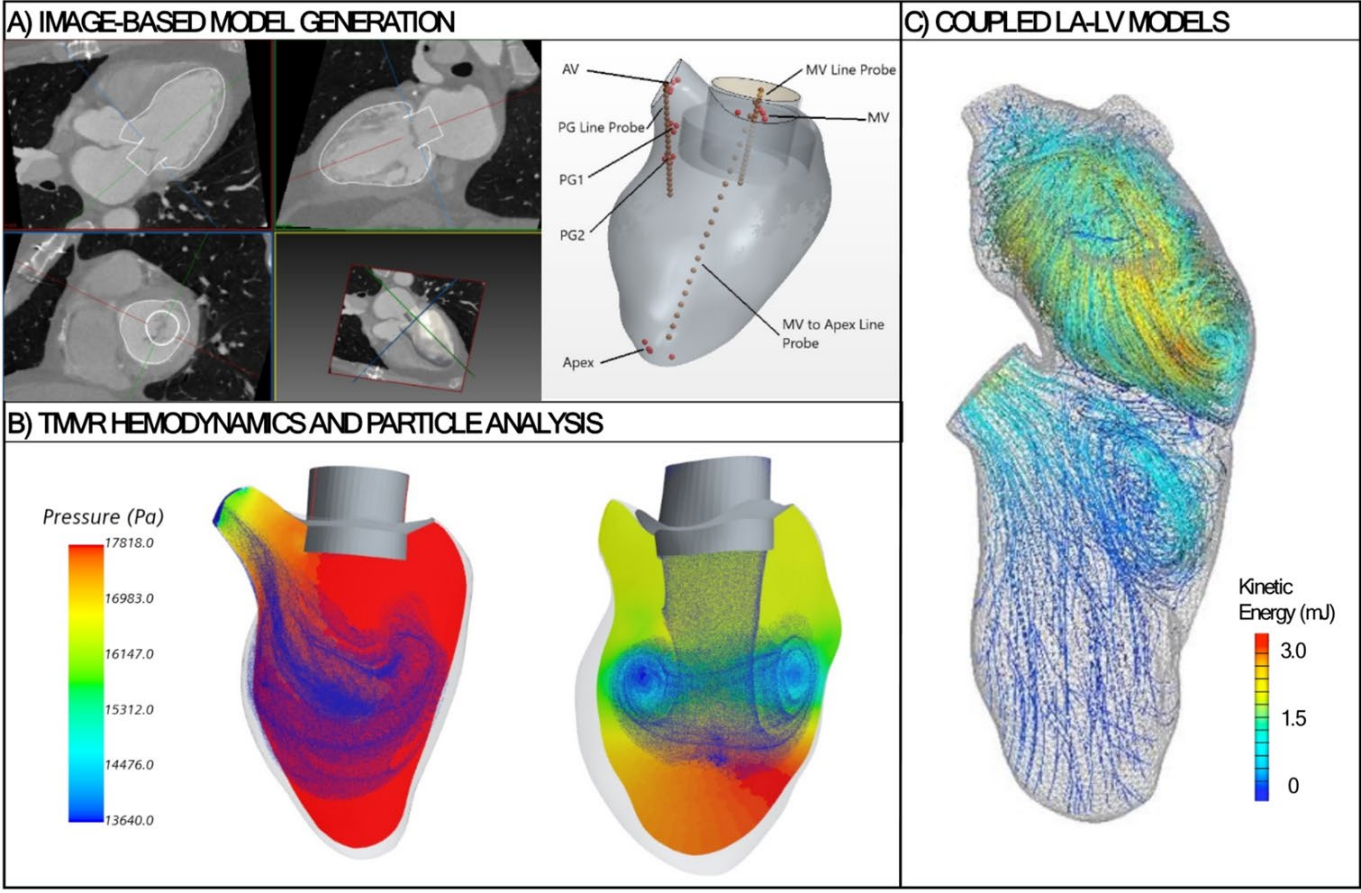
The application for computational flow dynamics in TMVR simulations to assess the risk of neo-LVOT obstruction. **A**) Manual segmentation of the left ventricle and measurement of the aorto-mitral angles. **B**) Pressure vectors in the left ventricle and neo-LVOT during systole and diastole after TMVR, with velocity of the particles represented from low (blue) to high (red). **C**) Streamlines and vortex structures in coupled models of left atrium and left ventricle, with velocity fields represented from low (blue) to high (red). (AV: aortic valve; LA: left atrium; LV: left ventricle; LVOT: left ventricular outflow tract; MV: mitral valve; PG: peak gradient; TMVR: transcatheter mitral valve replacement.)

The growing experience with pre-procedural planning for TMVR suggests that the estimation of neo-LVOT based on a single cardiac phase may be too simplistic and that further refinements may be required. This is particularly relevant when a binary cut-off for the neo-LVOT area is used to predict outflow tract obstruction and screening failure rates for patients being considered for TMVR remain high. Outflow tract obstruction is a complex physiological phenomenon and does not have a linear relationship to the geometric neo-LVOT area. It relies on multiple factors that include the geometry of the left ventricle, the mitral valve annulus, the aorto-mitral valve angulation and pressure within the left ventricle. Furthermore, functionally significant and clinically relevant LVOT obstruction can occur anywhere between early-, mid- and end-systole, which underpins the recommendations to perform a multi-phase MDCT when evaluating eligibility for TMVR (Fig. 4) [51, 52]. Early work also indicates considerable promise in using CFD models based on pre-procedural dynamic MDCT scans as an alternative method to predict the risk of neo-LVOT obstruction following TMVR (Fig. 5) [28, 49, 53–56]. 

While TTVR is achieving increased global interest, there is limited data on computational modelling in this space at present, although efforts are underway. The marked separation and the wide-open angle between the tricuspid valve and the pulmonary valve make the risk of right ventricular outflow tract (RVOT) obstruction negligible with any type of tricuspid valve intervention [57]. Apart from the repair techniques using tricuspid valve leaflet edge-to-edge devices, there are now a multitude of transcatheter tricuspid valve replacement (TTVR) options [58]. These are broadly categorised as orthotopic, where the prosthesis is placed in the tricuspid valve annulus, and heterotopic, where valves are deployed in one or both vena cavae to diminish the haemodynamic consequences of tricuspid regurgitation (TR). The TTVR devices have the potential to fulfil the unmet clinical need of treating patients who are not anatomically suitable for repair due to large coaptation gaps, short and/or extremely tethered septal leaflets or complex tricuspid valve morphology with four or more leaflets [59]. Concerns regarding access route selection, residual regurgitation, coronary sinus compromise and interaction with the conduction system or pacing devices are vital for consideration when planning TTVR. Due to the complex and non-planar geometry, dynamic assessment of the tricuspid valve annulus during the entire cardiac cycle with multi-phase MDCT may help with optimal TTVR sizing [60]. Patient-specific simulations of the TTVR procedure could assist with determining the most appropriate valve for an individual, optimal placement of the device and access route compatibility for dedicated catheter and steering system delivery (Fig. 3C) [17]. Further research is warranted to evaluate the benefits of implementing rehearsal simulations on the safety and efficacy of transcatheter tricuspid valve interventions as well as the broader range of structural interventions such as left atrial appendage occlusion (Fig. 3D).

## Three-dimensional printing

Advanced MDCT image processing can produce digital 3D models for better appreciation of complex spatial relationships in structural cardiac abnormalities [61]. They can be viewed, manipulated and rotated in any orientation, allowing a more natural and functional view of individual patient’s anatomy. However, these images are still viewed largely on two-dimensional (2D) computer displays. More recently, the application of 3D printing has become increasingly popular for tangible appreciation of cardiac anatomy and the planning of structural heart interventions [62]. 

The process of 3D printing refers to the production of accurate patient-specific anatomical replicas by depositing materials in layers based on digitally defined geometries [63]. The creation of 3D printed models involves several stages, including acquisition of good quality and artefact-free volumetric images, data post-processing, print material selection and product manufacturing. Due to high spatial resolution and excellent soft tissue characterisation, MDCT has been the most commonly used imaging modality for 3D printing [64]. The post-processing stage uses specialised software for image segmentation to delineate the boundaries of the specific region of interest intended for 3D printing [65]. Once the segmentation is complete, it can be converted into a digital model for 3D printing. The widely used 3D printing techniques include fused deposition modelling, selective laser sintering, stereolithography and material jetting [66]. Recent advances in the simultaneous use of rigid and flexible 3D print materials as well as different colours have created opportunities for increasingly sophisticated biological tissue printing. These technological advances allow the simulation of approximate mechanical properties of cardiovascular tissues to assess patient-specific tissue-device interactions [67, 68]. 

**Fig. 6:**
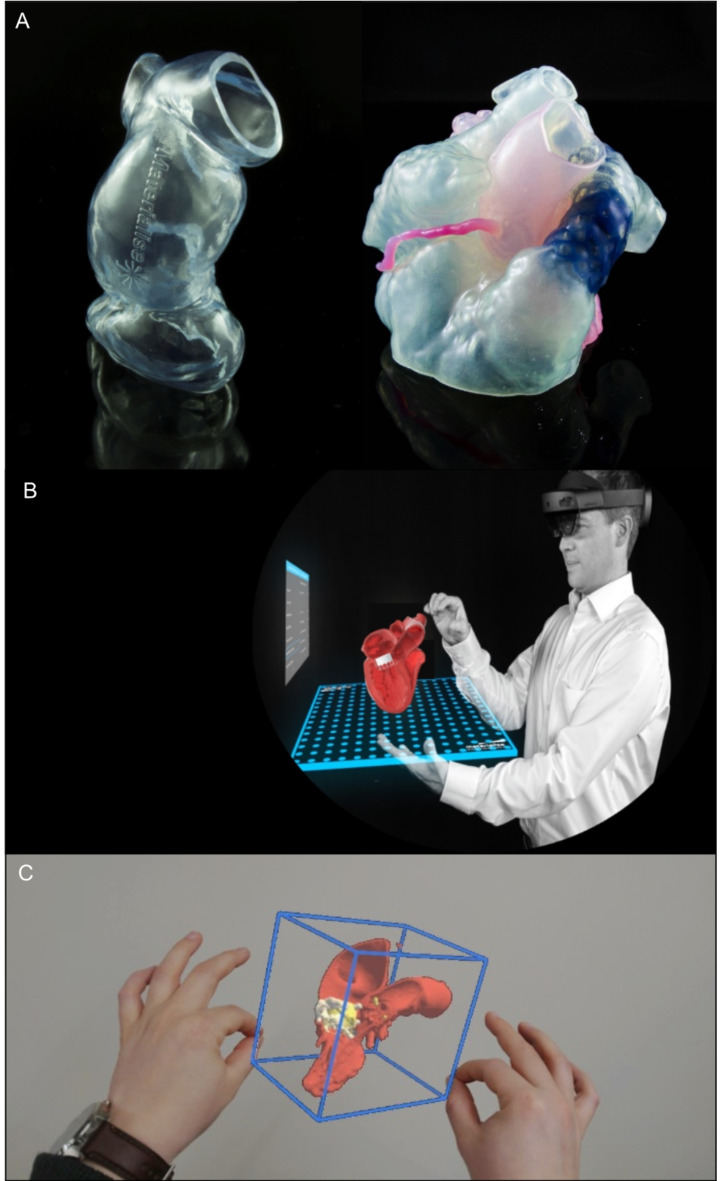
Examples of 3D printed heart models and application of extended reality for planning of structural heart interventions. **A**) 3D print examples of right ventricular outflow tract and pulmonary valve in a patient with congenital heart disease. **B-C**) Example of augmented reality analysis for improved visualisation in TMVR planning. (Mimics Enlight, Materialise, Leuven, Belgium). (TMVR: transcatheter mitral valve replacement; 3D: three dimensional.)

The tactile feedback from visual inspection of 3D models permits better appreciation of the cardiac anatomy for guiding challenging interventions and evaluating new technology [69]. Simulating the implantation of transcatheter devices into 3D models can assist with device type and size selection, refinement of procedural techniques and prediction of mechanical complications, thereby increasing procedural confidence [70]. This is particularly important for right heart interventions because of the inherent complexities of the tricuspid valve apparatus, the pulmonary valve and the RVOT in congenital heart disease (Fig. 6A) [71, 72]. Performing TAVR in vitro using the 3D printed models of the aortic valve is useful for predicting the risk of annulus rupture, coronary occlusion and significant PVL [73, 74]. The post-deployment haemodynamics within the sinuses of Valsalva can also be simulated using patient-specific anatomical replicas to evaluate the impact of TAVR on leaflet degeneration and valve thrombosis [75, 76]. Hands-on 3D printed models are suitable for benchtop simulations of transcatheter tricuspid as well as mitral valve edge-to-edge repair and replacement, helping to estimate the risk of post-procedural neo-LVOT obstruction or residual PVL [77, 78]. Applications in various other structural cardiac interventions have also been demonstrated, including left atrial appendage, PVL and atrial septal defect closure procedures [79]. Simulated interventions using patient-specific 3D models have been shown to reduce the learning curve for interventional operators, shorten the overall procedural time and decrease total radiation exposure, yielding a significant contribution towards the practice of precision medicine [19, 80]. Several notable disadvantages of 3D printing include limited ability to replicate the dynamic behaviour of the heart during the cardiac cycle, the mechanical properties of the cardiovascular tissues and the myocardial deformation after device deployment. The process for 3D printing can also be cumbersome and expensive ($55 - $810), requiring expert knowledge in multiple disciplines for successful implementation.

## Extended reality

Extended reality is an emerging medical imaging display platform, which encompasses virtual, augmented and mixed reality. More recently, the use of extended reality has attracted increasing attention and clinical application in structural cardiac interventions for more intuitive and immersive visualisations [81]. This technology can enhance the evaluation of complex 3D anatomical information and spatial relationships on pre-procedural MDCTs to improve the accuracy of procedural planning. In moving beyond the illusion of depth on the conventional 2D and 3D image displays, these tools create a transformative human-machine interface by providing the user with the possibility of real-time interaction with virtual 3D models in an alternative environment [82]. Technological improvements with the recent generation virtual reality headsets and powerful graphics cards have substantially improved the experience through more realistic images, enhanced depth perception and reduced motion sickness [83]. Mixed-reality visualisations can improve the confidence of the Heart Team during TAVR planning by enabling simulations of different device placement scenarios to identify difficult coronary re-access or increased risk of significant PVL [84–86]. The exact location of the trans-septal puncture can be planned virtually for mitral valve interventions, potentially reducing procedure-related complications (Fig. 6B-C) [87]. To take advantage of different modalities simultaneously, fusion of MDCT reconstructions and catheterisation laboratory fluoroscopy images can offer additional benefits. This has the potential to create an interactive roadmap for intraprocedural guidance of transcatheter interventions, reducing device wastage, procedure time and contrast use [88]. Overlaying the aortic annulus with the fluoroscopic images during TAVR can identify the appropriate coaxial angulation for device deployment and the optimal depth of implantation [89]. With fusion imaging, the location of the paravalvular defects can be displayed on fluoroscopy to guide wire and closure device placement [90]. Further research is needed to better understand the benefits of these immersive technologies for guiding other structural cardiac interventions at all stages of care, including patient education, pre-procedural planning and intra-procedural visualisation [91]. 

## Artificial intelligence-based procedure planning

Artificial Intelligence (AI) is an emerging field that aims to create algorithms to carry out tasks that usually require human intelligence [92]. Deep learning is a branch of machine learning in which a complex multi-layered neural network learns representations of data automatically by transforming the input data into multiple levels of abstractions [93]. Supervised deep learning frameworks allow the construction of complex models for MDCT pattern recognition and auto-segmentation, which can be used for learning from extremely large datasets and subsequently performing automatic feature extraction [94]. The rapid development of AI technologies presents an opportunity for its application in structural intervention to automate MDCT image analysis and improve the speed and accuracy of procedural planning [95]. 

The workup of patients being considered for structural cardiac interventions is a complex and multi-parametric process. MDCT is the gold-standard imaging modality for guiding TAVR planning by performing a detailed anatomical assessment of the aortic valve annulus, aortic root and aorto-iliac vessels [96]. This facilitates appropriate patient selection, device sizing and positioning, coplanar fluoroscopic angle prediction and determination of high risk features associated with annular injury, coronary occlusion and vascular complications in advance of the procedure [14]. Similarly, MDCT is an essential imaging modality for planning transcatheter atrio-ventricular valve interventions. This involves annulus sizing, geometrical assessment of the device landing zone, its relationship to adjacent coronary arteries, the height and angle of valve leaflet tenting, as well as the dimensions of the atria. Currently, technical criteria for intervention are assessed by performing precise manual or semi-automatic measurements of specific cardiac and peripheral vascular structures by experienced and qualified clinicians. However, human scan interpretation requires considerable training and can be time-consuming as well as prone to inaccuracies due to inter-observer variability and human error, especially for inexperienced interpreters [97]. These challenges can result in a time delay and assessment errors, which can have an adverse impact on device selection and deployment strategies [98]. It is expected that the volume of TAVR procedures will grow by a further 4 to 10 fold over the next decade, potentially limiting access to prompt and adequate treatment in overburdened centres [99]. With increasing efficacy data, population growth and ageing, it is also expected that the volume of transcatheter atrio-ventricular valve interventions will increase considerably [100, 101]. Accordingly, there is substantial clinical interest in novel strategies to optimise patient management pathways in order to meet the high service demands and avoid excess morbidity and mortality on the procedural waiting lists. Consequently, the development of completely automated quantitative analysis of pre-procedural MDCT scans using AI algorithms could provide a solution by streamlining clinical workflows through consistent identification of relevant anatomical structures in a more time-efficient manner [102]. 

The feasibility of AI tools for fast and accurate TAVR planning is an area of active research, with several tools emerging in recent years. TAVR-PREP is a deep learning-based prototype for pre-TAVR MDCT assessment, which performs a fully automated workflow for image segmentation, landmark detection and measurement extraction [103]. The performance of this system to obtain key parameters related to the aortic annulus, LVOT, Sinuses of Valsalva, sinotubular junction and coronary ostial heights was validated in 200 patients undergoing TAVR planning MDCT. The TAVR-PREP algorithm measures were highly correlated with manual assessments performed by expert cardiologists for most parameters (Pearson correlations: 0.90–0.97), except for left and right coronary heights (Pearson correlations: 0.80 and 0.72, respectively). Similarly, the mean absolute relative error was within 5% for most measurements, apart from left and right coronary heights (11.6% and 16.5%, respectively). The difficulties with precise identification of coronary ostial heights may be related to uneven intensity levels of MDCT images due to calcification, imprecise identification of aortic valve cusp nadirs and the inherent margin of error associated with landmark detection. This could be due to the relatively small training dataset used for TAVR-PREP, which is an important factor given the complexity of the aortic root structures and variability in pathological states.

The FORSSMANN is another recently developed AI algorithm based on deep learning for entirely automated TAVR MDCT analysis in patients with both tricuspid and bicuspid aortic valve stenosis [104]. The FORSSMANN system was trained using a large dataset consisting of 800 MDCT scans to achieve increased robustness and adaptability with a diverse range of aortic root morphologies and extreme calcification conditions. The accuracy of the algorithm was determined in the internal and external validation datasets, which showed a small mean difference between the AI measurements and senior observers. The correlation coefficients ranged from 0.866 to 0.998 for all key anatomical structures, demonstrating the importance of comprehensive algorithm training for superior MDCT interpretation and precise multi-parametric assessment.

**Fig. 7:**
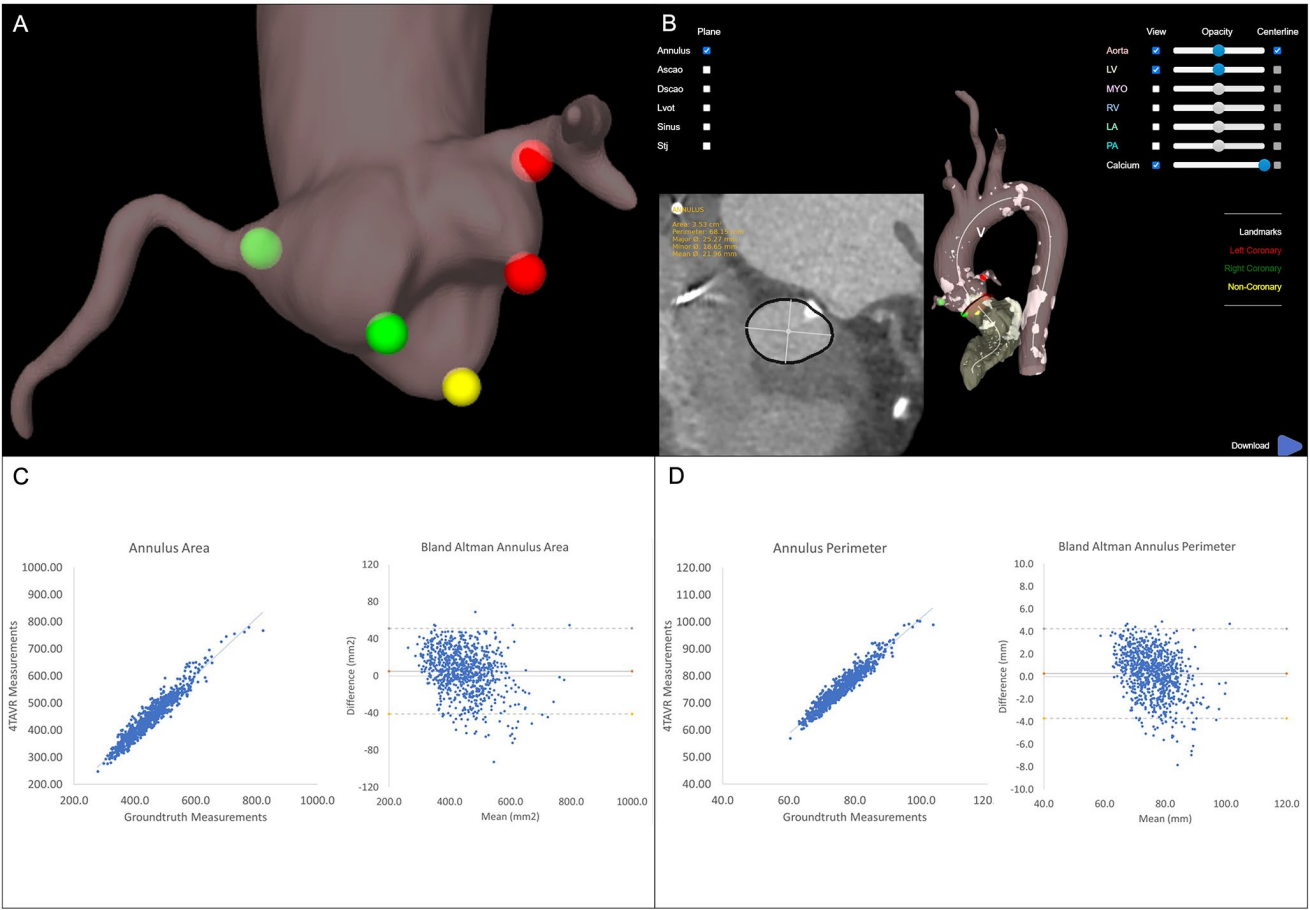
Illustration of fully automated artificial intelligence-based MDCT assessment for TAVR planning. **A**) Aortic root segmentation and automatic detection of aortic cusp insertion points and coronary artery ostia. **B**) Example of automatic aortic annular measurements in the context of basal annular calcification. **C**) Pearson correlation and bland-altman plots showing a strong agreement between the fully automated 4TAVR and manual measurements by experts for the aortic annulus area. **D**) Pearson correlation and bland-altman plots showing a strong agreement between the fully automated 4TAVR and manual measurements by experts for the aortic annulus perimeter. (4TAVR, Hi-D Imaging, Winterthur, Switzerland)(MDCT: multi-detector computed tomography; TAVR: transcatheter aortic valve replacement.)

Progressive technological advances have enabled AI systems for TAVR planning to be deployed as cloud-based platforms, with a potential to offer significant clinical and research value. 4TAVR (Hi-D Imaging, Winterthur, Switzerland) is the world’s first cloud-based AI software that provides a single-click solution to completely automated TAVR planning (Fig. 7). The overall 4TAVR analysis pipeline takes place over a set number of stages, including (1) deep learning-based segmentation, (2) deep learning-based detection of anatomical landmarks, (3) extraction of centre-line and profiling, (4) anatomical parameter assessment and (5) report generation. After the computations are completed, users can access clinically relevant anatomical measurements together with 2D and 3D reconstructed images in a viewer for optional manual adjustment, 3D-print ready segmentation meshes and interactive S-curves to access C-arm angles. To ensure substantial robustness and generalisability, the 4TAVR software was trained using 825 TAVR MDCT scans from three heart centres in the USA and Switzerland using different scanners and protocols. In an external validation study of 100 randomly selected TAVR MDCT scans, the AI aortic root measurements had excellent agreements with manual assessment by expert clinicians, with correlation coefficients ranging from 0.832 to 0.973 [105]. The added benefit of 4TAVR software is the ability to upload of multiple MDCTs for simultaneous analysis, thereby saving time and increasing hospital efficiencies.

**Fig. 8:**
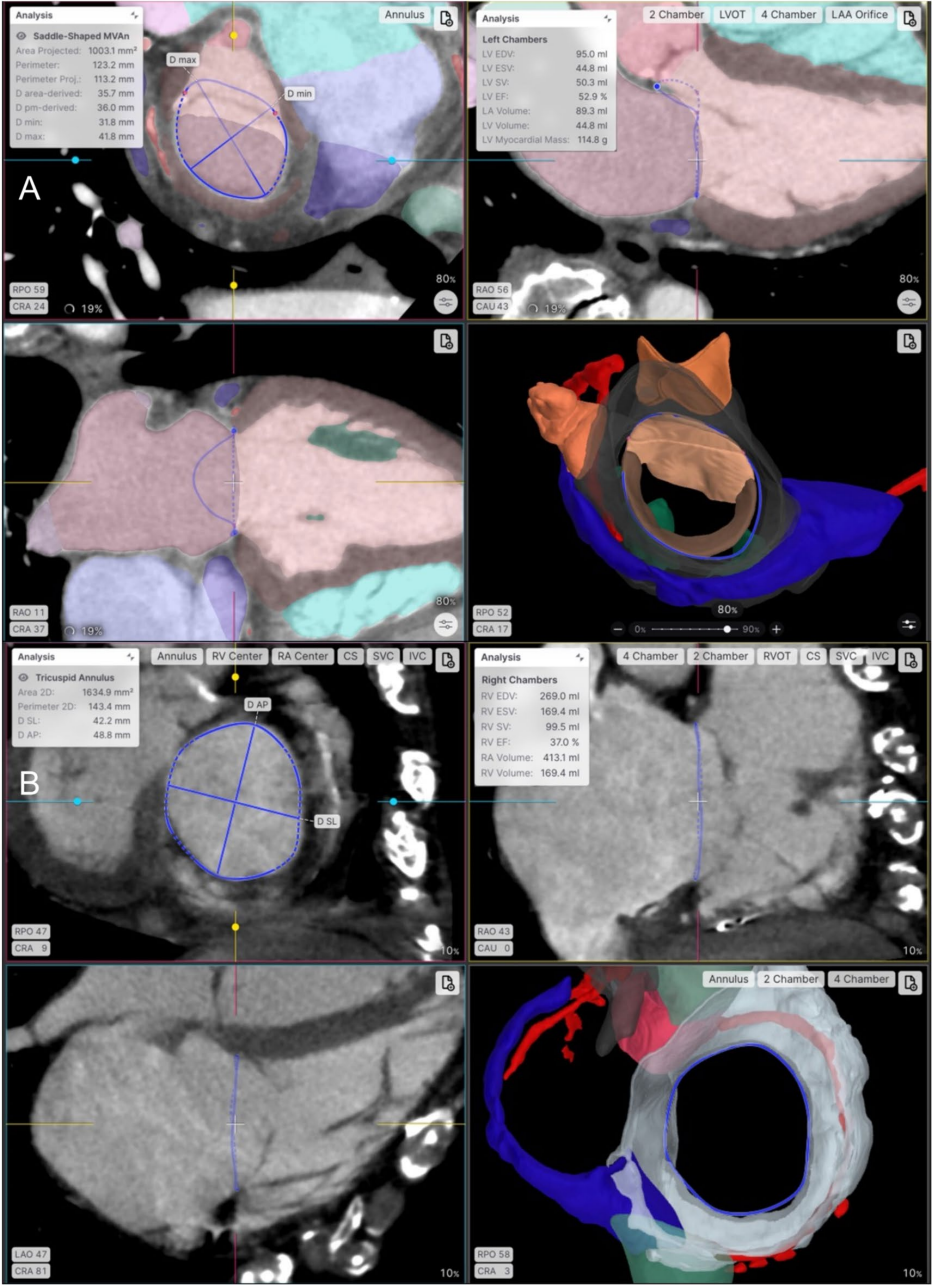
Demonstration of artificial intelligence-driven MDCT analysis for transcatheter atrio-ventricular valve interventions. **A**) Automatic functional and volumetric chamber analysis of the left ventricle, mitral annular sizing and 4D leaflet visualisation for mitral valve interventions. **B**) Tricuspid valve analysis with annular sizing, volumetric and functional measurements of the right ventricle, and overlaid segmentations. (Heart.ai, Laralab, Munich, Germany)

More recently, AI-based MDCT assessment has been applied to planning of mitral and tricuspid valve interventions. The Heart.ai (The LARALAB platform, Munich, Germany) is a deep learning-powered software, which provides a fully automated pipeline for MDCT analysis before transcatheter atrio-ventricular valve procedures (Fig. 8). The system generates a comprehensive set of segmentations that includes major structures such as the cardiac chambers, and finer structures down to the level of individual valve leaflets. Custom algorithms are used to automatically derive a wide range of measurements based on auto-segmentation, including anatomical, functional and volumetric parameters. The platform is available within a cloud-based viewer, which enables users to review the automatic segmentations, make final adjustments and generate reports. Although further research is required to assess the utility of this platform for risk-stratification and prognostication, early experience suggest that this technology is useful for pre-procedural decision-making and device selection, helping to identify patients at increased risk for procedural complexity [106, 107]. 

Overall, the strong agreement between AI algorithms and most expert manual measurements demonstrates the potential of this technology to support procedure planning decision making. However, the potential for publication bias in AI research should be noted, which could overestimate the validity and applicability of this technology. Systematic reviews and meta-analysis are essential to provide a more balanced and comprehensive assessment of AI in clinical practice. New systems should also be developed for femoral and alternative vascular access assessment, aiming to encompass the entire pre-procedural workflow. Additionally, AI algorithms should be refined to make suggestions about optimal device selection and size, thereby setting even higher standards in automated planning. Further work is also needed to examine the methods of integrating this technology into clinical workflows to improve real-world operational efficiency and reduce the cost of healthcare.

## Myocardial tissue characterisation using extracellular volume quantification

Myocardial fibrosis is a pathological remodeling process that affects the extracellular matrix of the heart in response to myocardial stress associated with valvular abnormalities [108]. These alterations can collectively impair cardiomyocyte contractility and relaxation, disturb regional nutrient and oxygen metabolism and interfere with electrical conductivity [109–111]. Invasive endomyocardial biopsy is the reference standard for quantification of myocardial fibrosis. However, this is associated with inherent procedural risks and limited diagnostic sensitivity [112]. As a result, there has been increased interest in the non-invasive methods for evaluating focal and diffuse myocardial fibrosis.

**Fig. 9:**
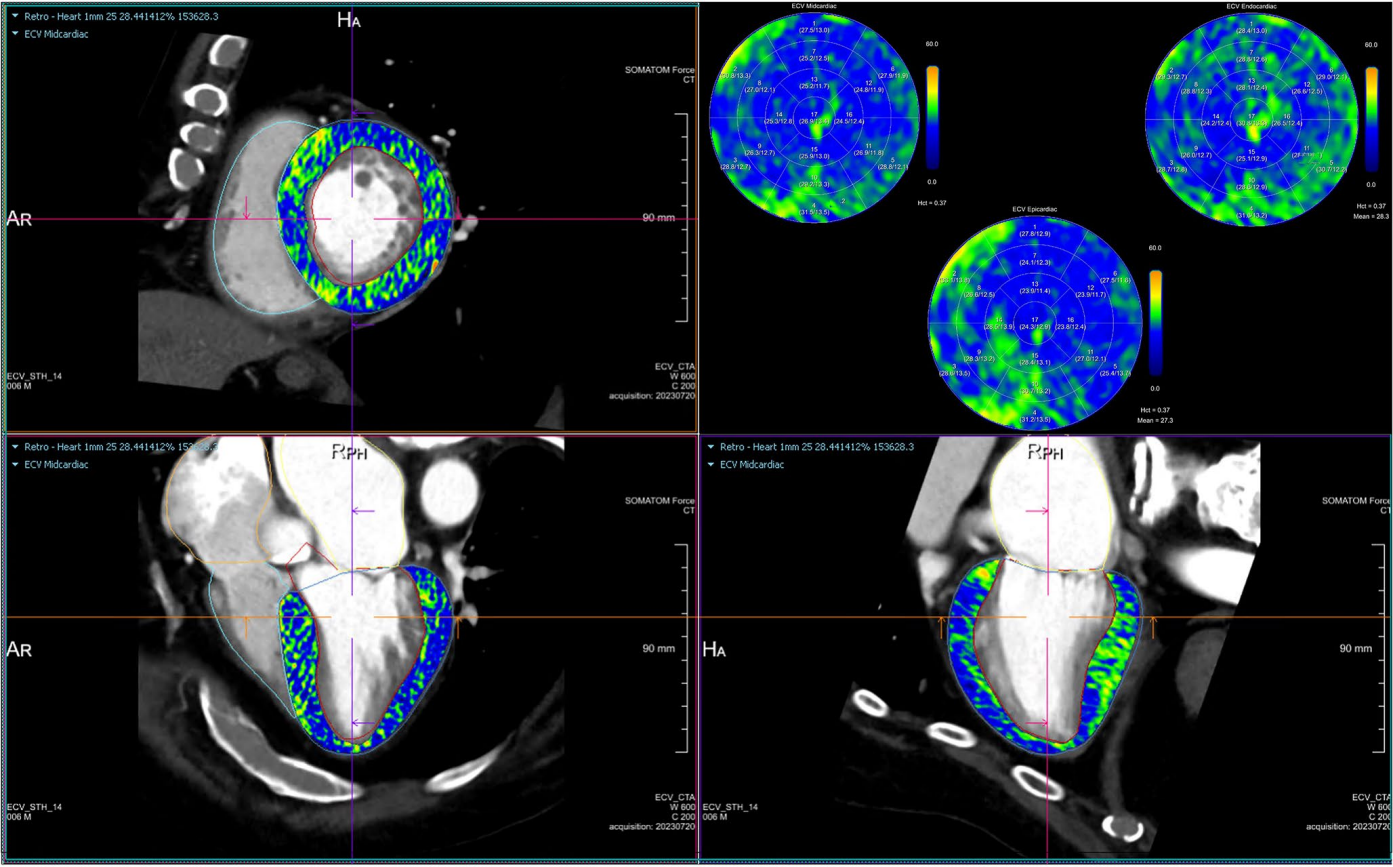
Extracellular volume images and polar maps calculated using the attenuation-based method from late enhancement MDCT images (CT Cardiac Functional Analysis, Siemens Healthcare, Germany). (MDCT: multi-detector computed tomography.)

CMR is well-established for myocardial tissue characterisation using both late gadolinium enhancement and extracellular volume (ECV) fraction quantification, which are highly correlated with biopsy-proven myocardial fibrosis and adverse clinical outcomes [113–116]. More recently, the introduction of low tube voltage and dual-energy scanning techniques has increased the MDCT contrast-to-noise ratio for improved myocardial tissue delineation. As a result, MDCT quantification of ECV (MDCT-ECV) has emerged as a robust and reliable alternative to CMR (Fig. 9) [117]. In comparison with CMR, MDCT is faster and cheaper, more widely available, achieves higher spatial resolution and required no additional considerations in patients with metallic implants or devices [118]. As a result, despite several disadvantages of MDCT related to ionising radiation, potentially nephrotoxic contrast agents and the lower contrast resolution compared to CMR, MDCT-ECV may offer a wider scope for risk prediction and prognostication in patients undergoing structural valvular interventions.

The ECV can be estimated using both single-energy or dual-energy MDCT scanners. The calculations method is based on the ratio between the contrast medium concentration in the myocardium and the plasma part of the blood pool [119]. Assessment of the haematocrit (Ht) is essential to convert whole blood to plasma concentrations. For single-energy MDCT-ECV calculation, an ECG-gated non-contrast enhanced scan is acquired first, followed by an ECG-gated late contrast enhancement scan at the equilibrium phase 3–10 min after contrast administration. The contrast injection protocol is usually adapted to administer 50-100mL or 1.4–1.8 mL/kg of contrast media to achieve sufficient contrast-to-noise ratio on late enhancement scans. The attenuation-based method measures ECV fraction by calculating the difference in Hounsfield units (∆HU) between the baseline and post-contrast administrations scans as follows:

$$\mathrm{ECV}=(1-\mathrm{Ht}) \times\left(\Delta H U_{\text {myocardium }} / \Delta H U_{\text {blood-pool }}\right)$$

More recently, dual-energy MDCT scanners have been used to derive the myocardial ECV using only the delayed contrast-enhanced acquisition, which results in lower radiation dose to the patient [120]. Dual-energy systems can be operated with different tube voltages or energy-sensitive detectors, leading to maximum spectral separation and optimised material differentiation for enhanced myocardial tissue characterisation [121]. This enables the creation of iodine maps by replacing true baseline non-contrast acquisitions with virtual non-contrast reconstructions [122]. Using this spectral method for ECV calculation, the ∆HU can be quantified directly from the iodine maps as follows:

$$\text { ECV }=(1-\mathrm{Ht}) \times\left(\text { lodine }{ }_{\text {myocardium }} / \text { Iodineblood-pool }\right)$$

Previous validation studies have demonstrated a strong correlation between MDCT-ECV, CMR-ECV and histological markers of extracellular fibrosis in animals models and patients with cardiovascular disease [123–127]. The prognostic role of MDCT-ECV has been widely demonstrated in patients with aortic stenosis, where increased ECV was associated with markers of functional status, B-natriuretic peptide, recovery of left ventricular systolic function and increased risk of death or heart failure hospitalisation after TAVR [128–131]. MDCT-ECV may help to screen for previously unrecognised cardiac amyloid in patients with severe AS scheduled for TAVR [132]. This is particularly important in this cohort since MDCT is the gold standard imaging modality for TAVR planning and the incidence of cardiac amyloid is increased in aortic stenosis.

## Clinical perspective

The developments in MDCT image processing techniques hold substantial potential for advancing transcatheter valvular interventions by offering the means for powerful computational analyses and efficient data processing. However, several important challenges must be addressed to ensure their safe integration into clinical workflows alongside the irreplaceable clinical judgment of experienced physicians. First, large scale randomised controlled trials comparing the clinical outcomes with the novel methods for procedure planning and the current standard of care are required. Second, to achieve the goal of patient-cantered treatment planning, all available transcatheter valves should be incorporated into the simulation and computational modeling platforms to predict the relevant device-host interactions. Third, to achieve greater generalisability and accuracy of AI models, more robust development will be required with access to large volumes and high-quality training datasets. Future collaborations between the developers and healthcare professionals will be needed to ensure effective evaluation of these techniques to demonstrate real-world validity, reproducibility, usability and reliability. Fourth, the application of these technologies for planning more complex procedures, including valve-in-valve and valve-in-ring interventions, should be carefully examined. Finally, to demonstrate the economic viability of these tools to support clinical decision making and procedure planning, detailed cost-effectiveness analyses are required. This is particularly important since some of these techniques may necessitate a degree of manual image processing, long computational times, extensive measurement adjustment or expensive subscription charges, which could render them clinically impractical.

## Limitations

For the purposes of this review, the authors have referred to technologies that illustrate new approaches to MDCT that they have first-hand experience with. A host of other software and image processing companies also co-exist that incorporate to a lesser or greater degree similar approaches to handling cross sectional imaging datasets. The authors accept that individual user experience is required to gain confidence in these novel approaches to image interpretation. Furthermore, some will require further validation to determine whether they result in meaningful improvements to patient clinical outcomes.

## Conclusions

Multimodality cardiovascular imaging performs a broad range of roles for structural valvular interventions, including morphological diagnosis, prediction of prognosis and peri-procedural guidance. The rapid developments of MDCT technologies have been instrumental in improving the work-up of patients undergoing catheter-based valvular treatment. Advanced analytics and virtual simulations using patient-specific modelling and CFD have created the potential for detailed procedural planning and improvement in clinical outcomes through prediction of procedure-related compilations. 3D printing and extended reality use MDCT data to improve the visualisation of complex cardiac anatomy for accurate planning of interventions. Increased application of AI-based algorithms is unlocking new opportunities for precise, efficient and automated MDCT assessment, which will likely have a significant impact on standardising the planning of structural heart disease interventions in the future. Improved tissue characterisation using MDCT-ECV has the potential to assess the downstream remodelling effects of valvular heart conditions. The application of these techniques can provide unparalleled anatomical insights into the increasingly complex and diverse range of structural valvular interventions. Further research is needed to better delineate the role of these advanced methods in everyday clinical practice.

## Data Availability

No datasets were generated or analysed during the current study.
